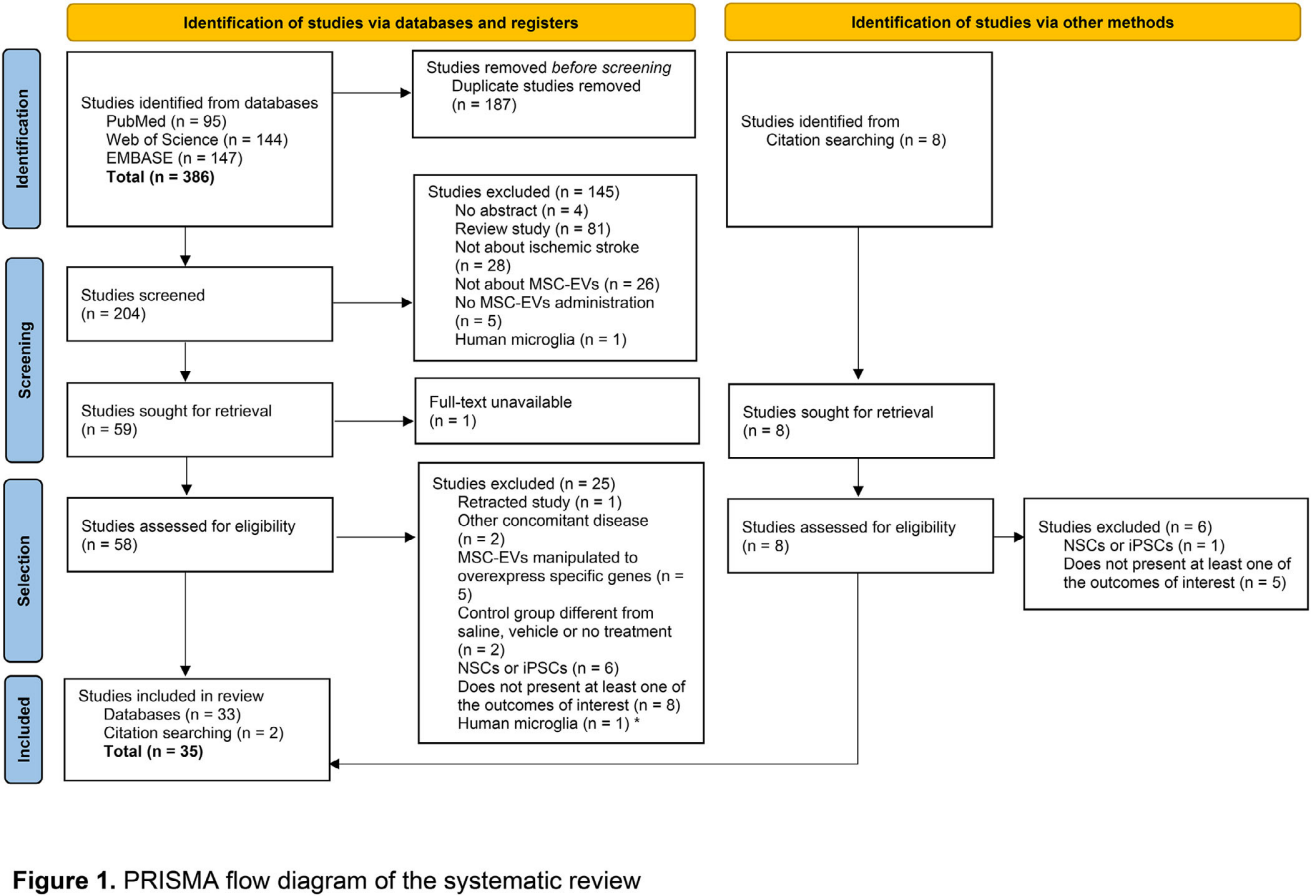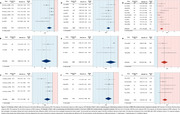# Early and Late Microglial Responses to MSC‐EVs in Animal Models of Ischemic Stroke: A Systematic Review and Meta‐Analysis

**DOI:** 10.1002/alz70855_103354

**Published:** 2025-12-23

**Authors:** Luis Pedro Bernardi, Thomas Hugentobler Schlickmann, Giovanna Carello‐Collar, Marco Antônio De Bastiani, Eduardo R. Zimmer, Francieli Rohden, Diogo O. Souza

**Affiliations:** ^1^ Universidade Federal do Rio Grande do Sul, Porto Alegre, Rio Grande do Sul, Brazil; ^2^ Universidade Federal do Rio Grande do Sul, Porto Alegre, RS, Brazil; ^3^ Brain Institute of Rio Grande do Sul (InsCer), PUCRS, Porto Alegre, Rio Grande do Sul, Brazil; ^4^ McGill Centre for Studies in Aging, Montreal, QC, Canada

## Abstract

**Background:**

Ischemic stroke (IS) is a risk factor for Alzheimer's disease (AD). In this context, microglial reactivity, a cellular response present in both conditions, has emerged as a promising target for therapeutic development. In a previous meta‐analysis, we showed that mesenchymal stem cell‐derived extracellular vesicles (MSC‐EVs) modulate microglial reactivity in animal models of IS. However, microglial responses dynamically change during early and late phases of IS. Thus, we aimed to investigate whether the effects of MSC‐EVs on microglia are different depending on disease phase.

**Methods:**

The meta‐analysis protocol was registered in PROSPERO (CDR42023463152) and followed the PRISMA 2020 statement. We searched EMBASE, PubMed, and Web of Science until January 2025 for studies assessing microglial reactivity following MSC‐EVs treatment in animal models of IS. We calculated standardized mean differences (SMD) via an inverse‐variance weight random‐effects model using the *metaviz* and *metafor* packages in R. Subgroup analyses were performed based on IS phases, defined as early (48–72h post‐ischemia) and late (≥168h).

**Results:**

Database search identified 386 studies, of which 35 were included (Figure 1). MSC‐EVs significantly reduced the number of Iba1+ cells and Iba1+ cells co‐expressing the pro‐inflammatory markers CD16, CD32, and iNOS in both early (SMD = ‐2.85 [95% CI: ‐4.12, ‐1.57], *p* <0.001; SMD = ‐1.98 [95% CI: ‐3.46, ‐0.51], *p* = 0.008; Figure 2B and 2E, respectively) and late (SMD = ‐1.07 [95% CI: ‐1.74, ‐0.40], *p* = 0.002; SMD = ‐1.41 [95% CI: ‐2.31, ‐0.51], *p* <0.001; Figure 2C and 2F, respectively) subgroups. Conversely, MSC‐EVs significantly increased the number of Iba1+ cells co‐expressing the anti‐inflammatory markers Arg‐1 and CD206 in both early (SMD = 2.01 [95% CI: 1.02, 3.00], *p* <0.001; Figure 2H) and late (SMD = 1.14 [95% CI: 0.56, 1.72], *p* <0.001; Figure 2I) subgroups.

**Conclusion:**

We demonstrated that MSC‐EVs consistently decreased pro‐inflammatory and increased anti‐inflammatory microglial responses in animal models of IS regardless of disease phase. These findings indicate that MSC‐EVs exert persistent modulatory effects on microglia, potentially contributing to the recovery of damaged brain tissue in IS and, consequently, in AD.